# The BTLA/HVEM axis as a key regulator for stratified functional cure in chronic hepatitis B

**DOI:** 10.3389/fimmu.2026.1882879

**Published:** 2026-08-10

**Authors:** Yuzhen Zhang, Shuqi Yang, Yanyan Lin, Yanyan Qiu, Xueping Yu

**Affiliations:** 1 Department of Infection Disease, Fujian Provincial Specialty Center for Liver Cirrhosis, Clinical Medical Research Center for Bacterial and Fungal Infectious Diseases of Fujian province, Fujian Medical University Affiliated First Quanzhou hospital, Quanzhou, Fujian, China; 2 Key Laboratory of Screening and Control of Infectious Diseases (Quanzhou Medical College), Fujian Provincial University, Quanzhou, Fujian, China

**Keywords:** BTLA, HVEM, chronic hepatitis B, T-cell exhaustion, immune checkpoint, stratified immunotherapy, soluble BTLA, functional cure

## Abstract

**Background:**

Functional cure rates in chronic hepatitis B (CHB) remain below 10% after long-term nucleos(t)ide analogue (NA) therapy, and PD-1/PD-L1 blockade achieves HBsAg clearance in approximately 30% of selected patients with low baseline HBsAg (≤100 IU/mL). This shortfall reflects an oversimplified view of T-cell exhaustion that neglects nonredundant checkpoints operating in parallel with PD-1.

**Main arguments:**

We synthesize emerging evidence (2019–2025) to position the B and T lymphocyte attenuator (BTLA)/herpesvirus entry mediator (HVEM) axis as an emerging candidate hub for layered therapeutic strategies in HBV infection. BTLA preferentially recruits SHP-1 (versus PD-1-predominant SHP-2) and signals via PI3K–AKT to sustain terminal exhaustion independently of PD-1 status. BTLA preferentially recruits SHP-1 (Src homology region 2 domain-containing phosphatase-1) [versus PD-1-predominant SHP-2 (Src homology region 2 domain-containing phosphatase-2)] and signals via PI3K–AKT to constrain TPEX proliferation and effector differentiation independently of PD-1 status. In CHB, BTLA is upregulated on peripheral and intrahepatic CD4+ and CD8+ T cells, correlating with ALT/AST and histologic activity; however, this correlation should be interpreted cautiously, as elevated liver enzymes primarily reflect immune-mediated hepatocellular injury during active inflammation rather than purely antiviral immune dysfunction. In HBV-related acute-on-chronic liver failure (HBV-ACLF), CD4+ T-cell BTLA expression independently predicts 90-day mortality and secondary infections. Soluble BTLA has emerged as a candidate minimally invasive biomarker for risk stratification in resource-limited settings.

**Conclusion:**

Under low antigen load following long-term NA suppression (HBsAg <100 IU/mL), biomarker-guided combination therapy targeting BTLA/HVEM alongside PD-1 may expand functional cure rates beyond the ceiling of single-checkpoint blockade. We outline an adaptive Phase I trial concept with predefined stratification thresholds to enable reproducible translation in HBV-endemic regions.

## 1 Introduction

Chronic hepatitis B virus (HBV) infection affects approximately 296 million people globally, causing 820,000 annual deaths from cirrhosis and hepatocellular carcinoma (1, 2). Despite potent viral suppression with nucleos(t)ide analogues (NAs), functional cure—sustained HBsAg loss—remains below 10% even after 5–10 years of therapy (3). The persistence of covalently closed circular DNA (cccDNA) and integrated viral genomes drives chronic antigen stimulation, leading to progressive T-cell exhaustion that constitutes the core biological barrier to functional cure (4). While T-cell exhaustion has traditionally been viewed as the central immunological barrier to functional cure, recent perspectives emphasize that HBV-specific CD8+ T-cell dysfunction encompasses features beyond classical exhaustion, including metabolic insufficiency, epigenetic reprogramming, and microenvironmental suppression (5). Within this broader landscape, checkpoint receptor upregulation, including BTLA, remains a clinically actionable component.

T-cell exhaustion in CHB follows a hierarchical progression underpinned by distinct cellular states with differential therapeutic responsiveness. Early exhaustion is PD-1-dominant and maps to a TCF1-high stem-like progenitor exhausted T-cell (TPEX) population that retains proliferative and differentiation capacity and constitutes the primary cellular substrate for PD-1 blockade. The T-cell factor 1 (TCF1)-high stem-like progenitor exhausted T-cell (TPEX) population retains proliferative and differentiation capacity and constitutes the primary cellular substrate for PD-1 blockade (6). Notably, TPEX cells persist throughout chronic infection and are not confined to an early temporal stage. Under persistent antigen stimulation, however, these TPEX cells progressively differentiate into terminally exhausted T cells (TEX) characterized by high thymocyte selection-associated high mobility group box (TOX) expression, epigenetic fixation of exhaustion programs, and permanent loss of proliferative capacity. Once this terminal state is reached, cells become refractory to PD-1 monotherapy because the underlying epigenetic landscape, rather than merely surface checkpoint expression, precludes functional recovery. Intermediate exhaustion adds TIM-3, requiring dual inhibition. Terminal exhaustion is marked by a PD-1+TIM-3+BTLA+ triple-positive phenotype with epigenetic fixation and absent proliferative capacity Intermediate exhausted subsets add T-cell immunoglobulin and mucin-domain containing-3 (TIM-3), where dual inhibition may be beneficial. Terminal exhaustion is marked by a PD-1+TIM-3+ phenotype with epigenetic fixation and absent proliferative capacity (7). In this deepest exhaustion state, BTLA is markedly upregulated on TOX-high terminally exhausted CD8+ T cells and on regulatory-like CD8+BTLA+ subsets enriched in hepatic parenchyma. This terminal subset remains refractory to PD-1/TIM-3 dual blockade because persistent BTLA-mediated suppression via preferential SHP-1 recruitment sustains dysfunction independently of PD-1 status BTLA is highly expressed on TPEX cells but is downregulated upon terminal differentiation (8–10). In the hepatic microenvironment, a regulatory-like CD8+BTLA+ subset is enriched in hepatic parenchyma (11, 12). Persistent BTLA-mediated suppression via preferential SHP-1 recruitment can constrain TPEX proliferation and effector differentiation independently of PD-1 status (13, 14). Consequently, PD-1 blockade reinvigorates the progenitor pool but cannot rescue terminally exhausted cells, whereas BTLA blockade targets the terminal subset and may interrupt the TPEX-to-TEX differentiation trajectory, preserving the progenitor pool that PD-1 blockade relies upon. BTLA thus represents a potentially nonredundant target that may be required for reversing terminal exhaustion and may provide additive benefit beyond PD-1 monotherapy, pending direct interventional evidence in stable CHB (**Table 1**). Consequently, PD-1 blockade reinvigorates the TPEX progenitor pool but cannot rescue terminally differentiated TEX cells (6, 13). BTLA blockade, by contrast, may act on the TPEX compartment to enhance their proliferative output and differentiation toward effector-competent intermediates, thereby expanding the pool of PD-1-responsive cells (15, 16). BTLA thus represents a potentially nonredundant target that may provide additive benefit beyond PD-1 monotherapy, pending direct interventional evidence in stable CHB (**Table 1**).

**Table 1:** Evidence grading for BTLA/HVEM-targeted immunotherapy in chronic hepatitis B.

Level	Category	Key findings/claims	Principal evidence	Limitations
I	Direct CHB observational evidence	BTLA/HVEM upregulated on peripheral and intrahepatic CD4^+^/CD8^+^ T cells; correlates with ALT/AST and histologic activity; intrahepatic regulatory-like CD8^+^BTLA^+^ subset enriched in liver; BTLA/HVEM spatial co-localization in portal–sinusoidal niches BTLA/HVEM upregulated on peripheral and intrahepatic CD4+/CD8+ T cells; correlates with ALT/AST and histologic activity; intrahepatic regulatory-like CD8+BTLA+ subset enriched in liver; BTLA/HVEM spatial co-localization in portal–sinusoidal niches; PD-1 dominates inhibitory receptor hierarchy on HBV-specific CD8+ T cells; PD-1 blockade response linked to intermediate T-cell differentiation	Song 2022; Wang 2017; Panão-Costa 2025; Bengsch 2014	Association only; no interventional data; single-centre designs; small-to-moderate sample sizes; peripheral-intrahepatic concordance assumed but not prospectively validated; HLA-A2 restricted cohort
II	HBV-ACLF evidence	CD4^+^ BTLA expression independently predicts 90-day mortality and secondary infections; BTLA deficiency or neutralizing antibody reduces mortality in ACLF models; BTLA/HVEM markedly elevated in ACLF liver tissue	Yu 2024; Xu 2012	Critical illness population; limited generalizability to stable CHB; small ACLF sample (Xu 2012)
III	Cancer/non-HBV chronic inflammation evidence	BTLA+PD-1 dual blockade reverses CD8^+^ T-cell exhaustion in NSCLC; BTLA regulates γδ T-cell cytotoxicity, Tfh function, and NK-cell IFN-γ production	Zhang 2025; Hwang 2021; Mintz 2019; Sordo-Bahamonde 2021	Tumour/non-HBV microenvironments; species and disease heterogeneity; preclinical or non-HBV clinical models
IV	Preclinical mechanistic evidence	BTLA preferentially recruits SHP-1 vs PD-1-predominant SHP-2; differential phosphatase wiring via ITIM/ITSM; PI3K–AKT pathway sustains terminal exhaustion; HVEM multiligand topology (CRD1/CRD2/3) and cis/trans interactions	Xu 2020; Xu 2021; Liu 2021; Battin 2022	*In vitro* or structural studies; limited validation in primary HBV-specific T cells
V	Speculative translational hypotheses	Under low antigen load (HBsAg <100 IU/mL), biomarker-guided BTLA targeting ± PD-1 blockade may expand functional cure rates beyond single-checkpoint ceiling; three-tier stratification framework; adaptive Phase I trial concept	Present manuscript	No direct clinical trial data in NA-suppressed CHB; hypothetical dosing and biomarker thresholds; unvalidated companion diagnostic criteria

The B and T lymphocyte attenuator (BTLA, CD272) and its ligand herpesvirus entry mediator (HVEM, TNFRSF14) form a unique axis with multiligand topology, cis/trans interactions, and bidirectional signaling (17). Evidence prior to 2019 was largely descriptive. However, 2019–2025 investigations have yielded critical mechanistic and clinical insights, including a landmark multicenter study establishing a strong association between BTLA and HBV-related acute-on-chronic liver failure (HBV-ACLF) mortality, with mechanistic validation in experimental models (18, 19). No prior framework has integrated this evidence to define a therapeutic window or establish standardized criteria for clinical translation.

Before proceeding, we emphasize a critical distinction. The immunopathology of stable CHB under long-term NA suppression differs fundamentally from that of HBV-ACLF. Stable CHB is characterized by low antigen burden and progressive, potentially reversible T-cell exhaustion. HBV-ACLF, by contrast, is a state of hyperinflammation and immune paralysis driven by high antigen load and cytokine storm. While both states implicate BTLA, the therapeutic windows, risk profiles, and translational goals differ markedly. Herein, we focus primarily on BTLA-targeted strategies for NA-suppressed stable CHB, using ACLF evidence only to illustrate mechanistic extremes.

Here, we argue that the BTLA/HVEM axis should be positioned as an emerging candidate hub for stratified immunotherapy in CHB. We explain the mechanistic basis for single-checkpoint failure and provide an actionable roadmap for biomarker-guided trial design tailored to HBV-endemic Asia-Pacific regions.

## 2 Nonredundant topology and signaling

BTLA is an immunoglobulin superfamily co-inhibitory receptor expressed on T cells, B cells, dendritic cells, and NK cells. Notably, PD-1 and BTLA exhibit distinct expression kinetics across T-cell differentiation. PD-1 is minimally expressed on naïve T cells and is progressively upregulated following antigen stimulation, peaking under sustained chronic exposure. In contrast, BTLA is constitutively expressed on naïve and resting T cells and can further accumulate during chronic antigen stimulation. These temporal differences imply that BTLA predominantly regulates early activation thresholds and memory formation, whereas PD-1 acts as a dominant brake during effector differentiation and terminal exhaustion. Consequently, the two receptors control non-overlapping phases of the T-cell lifecycle, and dual blockade may be required to restore function across the full differentiation spectrum in CHB. Beyond these temporal differences, BTLA and PD-1 diverge markedly in their cellular distribution. While PD-1 is largely restricted to activated T cells, BTLA is additionally expressed on γδ T cells and myeloid-derived immune populations, including macrophages and dendritic cells (20). This broad expression profile suggests that BTLA/HVEM modulation may reshape multiple arms of hepatic immunity, including innate effector function, antigen presentation, and myeloid-cell regulation, beyond the T-cell-centric consequences of PD-1 blockade. HVEM, a TNFR superfamily member, possesses a unique multiligand architecture: its cysteine-rich domain 1 (CRD1) preferentially binds BTLA and cluster of differentiation 160 (CD160), while CRD2/3 engage the stimulatory ligand LIGHT (TNFSF14), enabling simultaneous binding of functionally distinct ligands on a single molecule (21, 22). This topology allows the axis to fine-tune immune responses in a context-dependent manner within the tolerogenic liver microenvironment.

**Figure 1:**
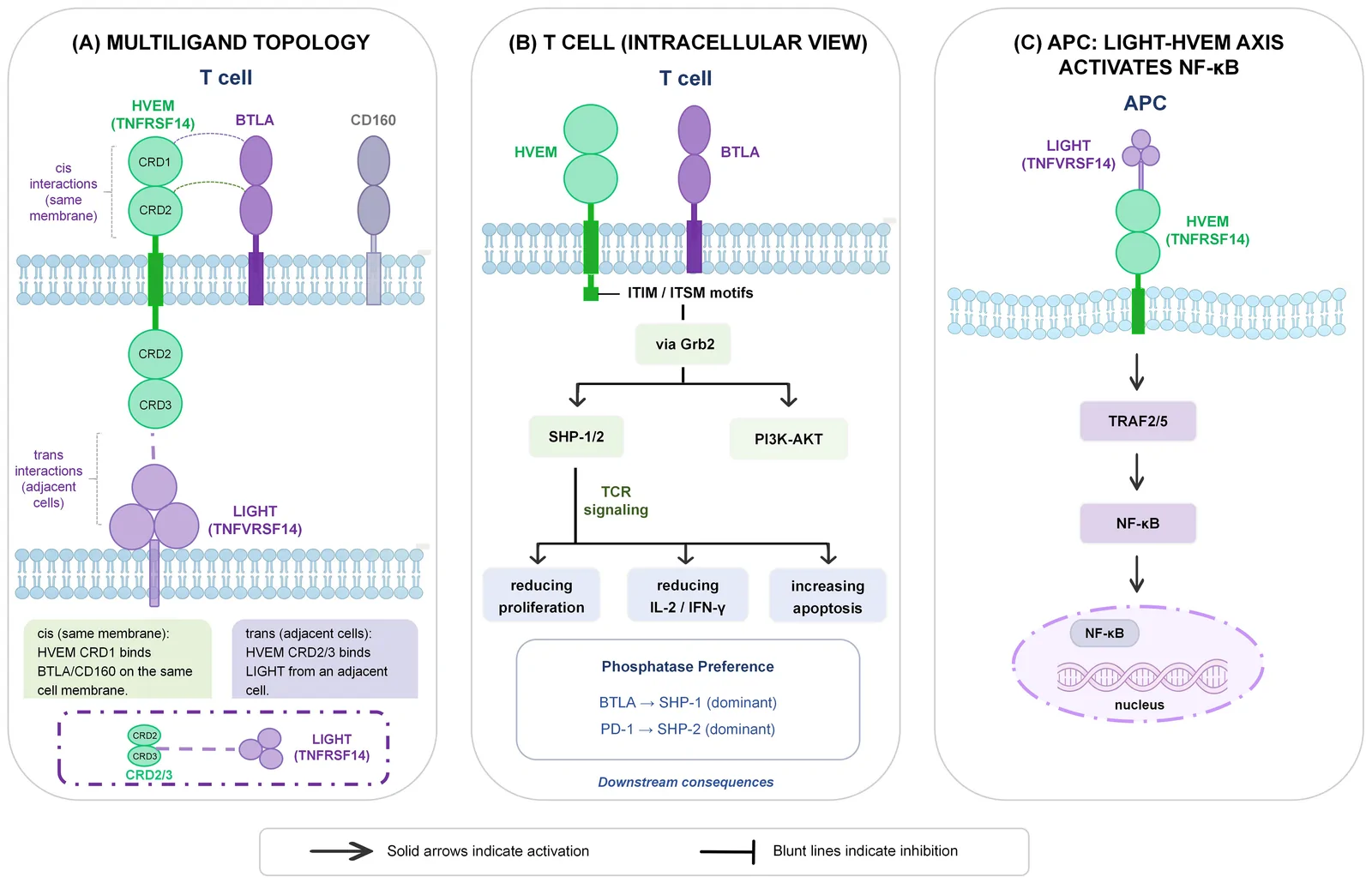
BTLA/HVEM pathway schematic and T-cell effects. **(A)** Multiligand topology on HVEM (TNFRSF14): CRD1 preferentially binds BTLA/CD160 and CRD2/3 bind LIGHT, enabling cis interactions (same membrane) and trans interactions (adjacent cells). **(B)** Intracellular signaling: ITIM/ITSM motifs recruit SHP-1/2 via Grb2, coupling to PI3K–AKT to attenuate TCR signaling, reducing proliferation and IL-2/IFN-γ and increasing apoptosis. **(C)** On APCs, LIGHT-bound HVEM signals via TRAF2/5 to activate NF-κB. Solid arrows indicate activation; blunt lines indicate inhibition. Schematic created with Microsoft PowerPoint and Procreate by the authors.

BTLA–HVEM interactions occur in cis (on the same cell membrane, forming dominant inhibitory complexes) or trans (between adjacent cells, mediating paracrine suppression) (23). The balance is dynamically regulated by inflammatory signals, creating a tunable regulatory axis in hepatic tissue (**Figure 1A**).

Intracellularly, BTLA contains an immunoreceptor tyrosine-based inhibition motif (ITIM) and an immunoreceptor tyrosine-based switch motif (ITSM) that recruit SHP-1 and SHP-2 via growth factor receptor-bound protein 2 (Grb2) upon engagement, coupling to the PI3K–AKT pathway. This dampens TCR signaling, reduces IL-2 and IFN-γ production, inhibits proliferation, and promotes apoptosis (**Figure 1B**).

A key feature contributing to PD-1 monotherapy failure is the differential phosphatase wiring between BTLA and PD-1 (13, 14). BTLA preferentially signals through SHP-1, whereas PD-1 predominantly couples to SHP-2, with only partial downstream overlap. PD-1 blockade releases SHP-2 inhibition but leaves BTLA–SHP-1 signaling largely intact, contributing to the therapeutic ceiling in BTLA-high populations. However, this phosphatase-centric model is incomplete: both receptors can suppress T-cell proliferation and cytokine production via SHP-1/SHP-2-independent mechanisms, including direct modulation of PI3K–AKT–mTOR signaling and metabolic reprogramming. In CHB, these parallel pathways likely cooperate with phosphatase-dependent circuits to sustain terminal exhaustion. Concurrent BTLA targeting may therefore be required to disable multiple suppressive circuits and restore function in terminally exhausted T cells. In CHB, these parallel pathways likely cooperate with phosphatase-dependent circuits to sustain terminal exhaustion. Concurrent BTLA targeting may therefore provide additive benefit by disabling suppressive circuits in the TPEX compartment, thereby enhancing progenitor expansion and effector differentiation beyond what PD-1 monotherapy achieves. This theoretical nonredundancy provides a rationale for investigating combination checkpoint blockade in CHB (16).

Beyond this T-cell-centric phosphatase and metabolic framework, BTLA exerts distinct regulatory effects across multiple immune compartments that collectively shape the liver microenvironment. On B cells and T follicular helper cells, BTLA engagement suppresses germinal-center reactions and antibody production; its blockade may therefore restore humoral immunity and promote anti-HBs antibody generation (24). On NK cells and γδ T cells, BTLA ligation dampens cytotoxicity and IFN-γ secretion, suggesting that BTLA-directed therapy could reactivate innate antiviral effectors that operate independently of classical T-cell recognition (25). On dendritic cells and other myeloid populations, BTLA-HVEM interactions impair antigen presentation and promote tolerogenic phenotypes; relieving this inhibition may enhance priming of *de novo* HBV-specific T-cell responses and reverse myeloid-mediated immune suppression within the hepatic niche (26). Thus, BTLA blockade offers the prospect of coordinated immune reconstitution spanning adaptive, innate, and antigen-presenting compartments—a breadth of action that exceeds the T-cell-centric profile of PD-1 inhibition.

In parallel, HVEM mediates bidirectional signaling: LIGHT-bound HVEM on antigen-presenting cells signals via TNF receptor-associated factor 2/5 (TRAF2/5) to activate NF-κB, promoting pro-inflammatory cytokine production and antigen presentation (**Figure 1C**). This dual capacity enables the axis to suppress excessive activation in the steady state while promoting inflammation during infection, making it a uniquely versatile regulator of hepatic immunity.

Spatial co-localization of BTLA/HVEM with PD-1/PD-L1 and TIM-3/galectin-9 (Gal-9) in portal–sinusoidal niches physically amplifies local immune suppression within the liver (27), suggesting that targeted intervention can enhance local antiviral immunity while minimizing systemic activation.

## 3 Clinical correlates and biomarker potential

Accumulating evidence demonstrates consistent BTLA upregulation on peripheral and intrahepatic CD4+ and CD8+ T cells in stable CHB, with levels correlating directly with ALT/AST, HBV DNA load, and histologic activity Accumulating evidence demonstrates consistent BTLA upregulation on peripheral and intrahepatic CD4+ and CD8+ T cells in stable CHB, with levels correlating directly with ALT/AST and histologic activity (28). It is important to note that elevated ALT/AST primarily reflects immune-mediated hepatocellular injury during active inflammatory phases rather than purely antiviral immune dysfunction; accordingly, BTLA upregulation in this context likely represents a compensatory inhibitory response to inflammatory cytokine signaling rather than a direct surrogate of T-cell exhaustion severity. Song et al. (28) further noted that BTLA/HVEM expression did not correlate with HBV DNA load, a finding consistent with the interpretation that BTLA upregulation tracks immune-mediated hepatic inflammation rather than viral replication per se. It is important to note that elevated ALT/AST primarily reflects immune-mediated hepatocellular injury during active inflammatory phases rather than purely antiviral immune dysfunction; accordingly, BTLA upregulation in this context likely represents a compensatory inhibitory response to inflammatory cytokine signaling rather than a direct surrogate of T-cell exhaustion severity. Intrahepatic HBV-specific CD8+ T cells exhibit high BTLA expression, Consistent with this inflammatory association, intrahepatic HBV-specific CD8+ T cells exhibit high BTLA expression, with a regulatory-like CD8+BTLA+ subset enriched in hepatic parenchyma (11, 12). The concordance between peripheral and intrahepatic compartments supports a peripheral-blood-first biomarker approach, reserving liver biopsy for mechanistic validation subsets.

Single-cell studies have clarified the hierarchical role of BTLA in terminal exhaustion. The PD-1+TIM-3+BTLA+ triple-positive subset represents the deepest exhaustion state, refractory to PD-1/TIM-3 dual blockade due to persistent BTLA–SHP-1 signaling (7, 13). Single-cell studies have clarified the hierarchical role of BTLA in T-cell exhaustion. BTLA is highly expressed on TPEX cells and is downregulated upon terminal differentiation (8–10, 13). Persistent BTLA–SHP-1 signaling in the TPEX compartment can constrain progenitor expansion and effector differentiation, and combined PD-1/BTLA blockade may reinvigorate TPEX cells to produce effector-competent TEX-int intermediates (15, 16). Beyond conventional T cells, BTLA also regulates γδ T-cell cytotoxicity, T follicular helper function, and NK-cell IFN-γ production (24, 25, 29). In the context of CHB, restoration of Tfh function may enhance B-cell help and anti-HBs antibody production, while revived NK and γδ T-cell activity provides complementary innate immune pressure against HBV-infected hepatocytes. Additionally, BTLA expressed on dendritic cells and myeloid populations can constrain antigen presentation and promote tolerogenic signaling; its blockade may therefore improve priming of *de novo* HBV-specific T-cell responses and remodel the intrahepatic immune-tolerant microenvironment beyond simple T-cell reinvigoration.

In HBV-ACLF, a state distinct from stable CHB, BTLA and HVEM are markedly elevated in liver tissue compared with stable CHB or healthy controls (18). CD4+ T-cell BTLA expression is markedly elevated and correlates with MELD and CLIF-SOFA scores. CD4+ T-cell BTLA expression is markedly elevated and correlates with Model for End-Stage Liver Disease (MELD) and Chronic Liver Failure-Sequential Organ Failure Assessment (CLIF-SOFA) scores. A landmark 2024 multicenter study established CD4+ BTLA as an independent predictor of 90-day mortality and secondary bacterial infections, with mechanistic validation showing that BTLA deficiency or neutralizing antibody administration reduces mortality and bacterial burden in ACLF models. Upstream drivers include interleukin-6 (IL-6)/signal transducer and activator of transcription 3 (STAT3) and TNF-α/NF-κB, which upregulate BTLA and initiate a feedforward cascade of inflammation → immune paralysis → adverse outcomes. These findings establish BTLA as a critical checkpoint in severe HBV-related inflammation. However, they reflect a hyperinflammatory, immune-paralytic state that differs mechanistically from the low-antigen exhaustion seen in stable CHB, and should not be directly extrapolated to functional cure strategies.

Soluble BTLA (sBTLA), detectable by standard ELISA, has emerged as a candidate minimally invasive biomarker in both stable CHB and HBV-ACLF, although its clinical interpretation differs by disease state. In HBV-ACLF, sBTLA predicts 90-day mortality independent of conventional severity scores (18). In stable CHB, sBTLA correlates with hepatic inflammatory activity and peripheral BTLA expression. Its minimally invasive nature makes it particularly suitable for resource-limited settings across the Asia-Pacific, where advanced flow cytometry or liver biopsy access is restricted Its minimally invasive nature makes it particularly suitable for resource-limited settings in HBV-endemic regions worldwide, where advanced flow cytometry or liver biopsy access is restricted (30, 31). To facilitate cross-study comparison and translational planning, we summarize the design, cohort characteristics, key findings, and limitations of the principal BTLA/HVEM investigations in HBV infection in **Table 2**.

**Table 2:** Summary of clinical and translational studies investigating the BTLA/HVEM axis in chronic hepatitis B virus infection.

Study (Year)	Cohort type/Study design	Sample size (n)	Disease stage	Cell type/Tissue analyzed	Key findings	Limitations
Xu et al. (2012) (12)	Retrospective IHC	4 HBV-ACLF, 16 CHB, 5 healthy	HBV-ACLF; CHB	Liver tissue (paraffin)	First report of intrahepatic BTLA/HVEM in HBV-ACLF; BTLA highly expressed in HBV-ACLF but absent in CHB/healthy; HVEM positive in ACLF, rare in CHB	Very small ACLF sample (n=4); single-centre; descriptive only; heterogeneous tissue sources (autopsy vs. biopsy)
Wang et al. (2017) (11)	Cross-sectional prospective	43 CHB, 20 healthy	CHB	PBMC and liver-infiltrating lymphocytes; HBV-specific CD8+ T cells	Intrahepatic HBV-specific CD8+ highly express BTLA; BTLA+CD8+ T cells are regulatory (IL-10+) and suppress effector T cells; BTLA correlates with HBV DNA and ALT/IFN-γ	Single-centre; small sample; no clinical outcome correlation; ex vivo assays
Song et al. (2022) (28)	Cross-sectional prospective	44 CHB, 40 healthy	CHB	Peripheral CD4+ and CD8+ T cells	BTLA and HVEM significantly upregulated on CD4+ and CD8+ T cells in CHB; expression correlates with ALT/AST but not HBV DNA; BTLA signaling inhibits T-cell proliferation and cytokine production	Single-centre; cross-sectional; no longitudinal data; causality not established
Yu et al. (2024) (18)	Multicentre prospective + experimental	PBMC: 90 NC, 104 CHB, 71 ACLF; LIL: 5 NC, 5 CHB, 7 ACLF	CHB; HBV-ACLF	Peripheral and intrahepatic CD4+/CD8+ T cells; DCs	CD4+ BTLA independently predicts 90-day mortality and secondary infections in HBV-ACLF; sBTLA predicts mortality independent of severity scores; HVEM upregulated on DCs; IL-6/STAT3 and TNF-α/NF-κB drive BTLA upregulation	ACLF-centric; CHB mainly comparator; needs external validation; rodent model translation requires confirmation
Panão-Costa et al. (2025) (27)	Retrospective IHC profiling	30 CHB (10 with HCC)	CHB	Liver tissue (FFPE)	BTLA (CD272) positive in >50% of CHB liver; localized in portal/sinusoidal inflammatory cells; expression strongly correlated with TIGIT; PD-1, TIM-3, and Gal-9 correlated with ALT	Single-centre; retrospective; descriptive only; no functional assays; HCC cases may confound expression
Bengsch et al. (2014) (6)	Cross-sectional prospective	98 HLA-A2+ CHB, 22 with tetramer responses	CHB	PBMC and intrahepatic lymphocytes; HBV-specific CD8+ T cells	PD-1 dominates inhibitory receptor expression (79.3%) on HBV-specific CD8+ T cells; PD-1 blockade produces strongest functional restoration (184% proliferation); response linked to intermediate T-cell differentiation (CD27+CCR7−CD45RA−); combination blockade inferior to PD-1 alone	HLA-A2 restricted; single-centre; *in vitro* assays only; inactive carrier bias

IHC, immunohistochemistry; PBMC, peripheral blood mononuclear cells; LIL, liver-infiltrating lymphocytes; NC, normal controls; FFPE, formalin-fixed paraffin-embedded.

## 4 A translational framework for stratified therapy

Before outlining the translational framework, we summarize the evidence base in **Table 1**, which grades the strength of supporting data across five levels. Levels I and II derive from human CHB and HBV-ACLF observational studies, respectively; Level III from cancer and non-HBV models; Level IV from *in vitro* and structural mechanistic studies; and Level V from speculative hypotheses requiring prospective clinical validation. Based on Level I to IV evidence, we propose a three-tier biomarker-driven framework for BTLA-targeted therapy in stable, NA-suppressed CHB as a translational hypothesis (**Table 1**). Evidence from HBV-ACLF is referenced only to illustrate mechanistic extremes of BTLA-mediated immune dysfunction; therapeutic strategies for ACLF require separate validation and are not addressed here.

To operationalize this framework, we propose a companion biomarker panel that prioritizes minimally invasive peripheral assessments for therapy initiation, reserving tissue-based evaluation for mechanistic validation or exploratory trial subsets. Cellular stratifiers include BTLA mean fluorescence intensity and positivity on peripheral and intrahepatic CD4+ and CD8+ T cells, and the frequency of PD-1+TIM-3+BTLA+ triple-positive exhausted subsets, which identify patients most likely to benefit from BTLA targeting. Fluid stratifiers encompass serum sBTLA and soluble HVEM (sHVEM), measurable via widely available ELISA platforms. Tissue stratifiers include spatial co-localization scores of BTLA with HVEM, PD-L1, and TIM-3 in portal–sinusoidal niches, quantified by multiplex immunofluorescence. Primary cellular stratifiers include BTLA mean fluorescence intensity and positivity on peripheral CD4+ and CD8+ T cells, together with the frequency of circulating PD-1+TIM-3+BTLA+ triple-positive exhausted subsets that identify patients most likely to benefit from BTLA targeting. Primary fluid stratifiers encompass serum sBTLA and soluble HVEM (sHVEM), both measurable via widely available ELISA platforms. Exploratory tissue stratifiers, reserved for research contexts to validate peripheral–intrahepatic concordance, capture spatial co-localization scores of BTLA with HVEM, PD-L1, and TIM-3 in portal–sinusoidal niches, quantified by multiplex immunofluorescence.

Tier 1: Low antigen burden (HBsAg <100 IU/mL). We prioritize this stratum for initial proof-of-concept trials because the safety margin is widest under long-term NA suppression. The therapeutic window is defined as HBsAg <100 IU/mL, undetectable HBV DNA, and minimal hepatic inflammation after ≥2 years of therapy. We emphasize that this threshold is intended as a proof-of-concept entry point for first-in-human BTLA-targeted trials, selected because the safety margin is widest rather than because it represents the sole eligible population. While BTLA expression is highest during active hepatic inflammation, the terminal exhaustion it sustains is a progressive, epigenetically fixed state that persists even after inflammatory activity resolves. Thus, NA-suppressed patients with minimal inflammation may still harbor residual HBV-specific T cells trapped in a PD-1+TIM-3+BTLA+ progenitor exhausted phenotype that is only partially responsive to PD-1 monotherapy. Moreover, in this low-antigen setting, HBV-specific CD8+ T cells retain a TCF1-high stem-like progenitor exhausted (TPEX/TMPC) phenotype (6, 32, 33), which constitutes the cellular substrate most amenable to checkpoint reinvigoration. In this setting, exhaustion is comparatively shallow; although checkpoint receptors are upregulated, epigenetic fixation remains incomplete. This creates an opportunity to restore antiviral T-cell function without excessive hepatotoxicity. From a safety perspective, this low-antigen stratum is expected to limit the absolute number of reactivated effector cells and thereby constrain the peak intensity of intrahepatic inflammation, reducing the likelihood of severe immune-mediated hepatitis or systemic cytokine release. The clinical benefit in this subgroup may include both acceleration of HBsAg clearance and an absolute increase in functional cure rates above the ~30% ceiling reported with PD-L1 monotherapy. While patients with HBsAg <100 IU/mL may exhibit a background rate of spontaneous decline, the decades-long natural history of CHB means that even acceleration of clearance carries substantial clinical and socioeconomic value. This contrasts fundamentally with HBV-ACLF, where high systemic inflammation and cytokine storm create a distinct risk-benefit profile. BTLA blockade in ACLF therefore requires more restrained, time-sequenced intervention in separately designed trials, and ACLF-derived biomarker thresholds should not be applied to stable CHB.

The choice between direct BTLA blockade and HVEM-targeted strategies depends on the desired depth and directionality of axis modulation. Anti-BTLA monoclonal antibodies directly interrupt BTLA→SHP-1 inhibitory signaling in T cells, offering the most potent reversal of terminal exhaustion, but carry a theoretical risk of disrupting cis-complex homeostasis on antigen-presenting cells and may potentiate systemic immune activation. By contrast, fragment crystallizable (Fc)-disabled soluble HVEM fusion proteins act as decoy receptors that sequester BTLA in trans without engaging Fc gamma receptor (FcγR)-mediated effector functions, thereby providing a more graded, dimmable suppression of inhibitory signaling while preserving HVEM→LIGHT co-stimulatory capacity on antigen-presenting cells. Anti-HVEM antibodies are reserved for contexts requiring dominant blockade of HVEM-mediated pro-survival signals in infected hepatocytes or stromal cells. For the NA-suppressed CHB setting, where the goal is to reawaken residual HBV-specific T cells without precipitating cytokine release syndrome or ALT flare, we prioritize anti-BTLA monoclonal antibody (mAb) as the primary investigational arm and HVEM-Fc as an alternative arm for patients with high baseline inflammatory markers such as ALT greater than 2 times the upper limit of normal (ULN) or elevated IL-6, given its more favorable safety profile in preclinical models. Preclinical studies demonstrate that BTLA and PD-1 dual blockade reverses CD8+ T-cell exhaustion more effectively than monotherapy (16), supporting the seamless expansion to combination cohorts described below.

The rationale for combining low-dose PD-1 blockade rests on the non-redundant phosphatase wiring established in Section 2. The rationale for combining low-dose PD-1 blockade with BTLA targeting rests on the non-redundant phosphatase wiring established in Section 2. BTLA preferentially recruits SHP-1, whereas PD-1 predominantly signals via SHP-2, with only partial downstream overlap. In terminally exhausted HBV-specific T cells, PD-L1 monotherapy releases SHP-2 inhibition but leaves the BTLA→SHP-1→PI3K-AKT circuit intact, explaining the approximately 30% functional cure ceiling. Conversely, BTLA monotherapy may be insufficient for early-exhausted subsets where PD-1→SHP-2 signaling remains dominant. Concurrent dual blockade therefore disables both inhibitory circuits, restoring TCR signal strength, IL-2 synthesis, and proliferative capacity more completely than either alone. In TPEX cells, where BTLA is highly expressed, PD-1 monotherapy releases SHP-2 inhibition but leaves the BTLA→SHP-1→PI3K-AKT circuit intact, potentially limiting the magnitude of progenitor reinvigoration (13, 14). Conversely, BTLA monotherapy may be insufficient where PD-1→SHP-2 signaling remains dominant. Although PD-1 blockade alone can restore function in TPEX populations in multiple settings (34, 35), concurrent dual blockade may further enhance TPEX proliferation and differentiation toward effector-competent intermediates by simultaneously disabling both inhibitory circuits (16). The low-dose PD-1 component is deliberately conservative to minimize the risk of immune-related hepatitis and cytokine release syndrome in the context of residual intrahepatic cccDNA, while still achieving sufficient SHP-2 pathway release to synergize with BTLA blockade. The low-dose PD-1 component is deliberately conservative to minimize the risk of immune-related hepatitis and cytokine release syndrome in the context of residual intrahepatic closed circular DNA (cccDNA), while still achieving sufficient SHP-2 pathway release that may synergize with BTLA blockade to enhance TPEX expansion and effector differentiation.

The therapeutic window is defined as low antigen burden following long-term NA suppression: HBsAg <100 IU/mL, undetectable HBV DNA, and minimal hepatic inflammation after ≥2 years of therapy. We emphasize that this threshold is intended as a proof-of-concept entry point for first-in-human BTLA-targeted trials, selected because the safety margin is widest rather than because it represents the sole eligible population. In this setting, exhaustion is comparatively shallow—checkpoint receptors are upregulated but epigenetic fixation remains incomplete—creating an opportunity to restore antiviral T-cell function without excessive hepatotoxicity. The clinical benefit in this subgroup may include both acceleration of HBsAg clearance and an absolute increase in functional cure rates above the ~30% ceiling reported with PD-L1 monotherapy. While patients with HBsAg <100 IU/mL may exhibit a background rate of spontaneous decline, the decades-long natural history of CHB means that even acceleration of clearance carries substantial clinical and socioeconomic value. This contrasts fundamentally with HBV-ACLF, where high systemic inflammation and cytokine storm create a distinct risk-benefit profile. BTLA blockade in ACLF therefore requires more restrained, time-sequenced intervention in separately designed trials, and ACLF-derived biomarker thresholds should not be applied to stable CHB.

Interventional strategies include humanized anti-BTLA antibodies, anti-HVEM antibodies, and Fc-disabled soluble HVEM fusion proteins, which enable graded modulation of BTLA signaling without provoking cytokine release syndrome (36–38). Preclinical studies demonstrate that BTLA and PD-1 dual blockade reverses CD8+ T-cell exhaustion more effectively than monotherapy (16), supporting the seamless expansion to combination cohorts described below. Because BTLA is expressed on B cells, NK cells, γδ T cells, dendritic cells, and myeloid populations in addition to conventional T cells, BTLA-directed therapies may simultaneously enhance humoral immunity, restore innate effector function, and improve antigen presentation—reshaping the liver immune-tolerant microenvironment through multiple parallel mechanisms rather than limiting effects to T-cell reinvigoration alone.

Given this broad cellular distribution, systemic BTLA blockade carries a distinct theoretical adverse-event profile that extends beyond the T-cell-centric toxicities characteristic of PD-1 inhibitors. On B cells and T follicular helper cells, loss of BTLA-mediated restraint may enhance germinal-center reactions and, in susceptible individuals, promote autoantibody production with risk of autoimmune-like endocrinopathies, cytopenias, or immune-complex-mediated tissue injury. On NK and γδ T cells, unleashed cytotoxicity and IFN-γ secretion could amplify intrahepatic inflammation and precipitate systemic inflammatory responses resembling cytokine-release syndrome. On dendritic cells and macrophages, relief of BTLA-mediated suppression may enhance antigen presentation and pro-inflammatory cytokine release, potentially intensifying T-cell priming beyond the liver and raising the risk of colitis, dermatitis, or pneumonitis. These multi-compartment risks imply that the safety margin of systemic anti-BTLA therapy may be narrower than that of PD-1 monotherapy and closer to that of dual checkpoint blockade. However, this broad spectrum immune restoration carries inherent safety liabilities. Immune related adverse events (irAEs) are a well-recognized limitation of checkpoint inhibitors: PD 1 monotherapy causes all grade irAEs in approximately 70% of patients and grade ≥3 toxicity in 14 to 21% Immune-related adverse events (irAEs) are a well-recognized limitation of checkpoint inhibitors: PD-1 monotherapy causes all-grade irAEs in approximately 70% of patients and grade ≥3 toxicity in 14–21% (39), while dual CTLA 4/PD 1 blockade escalates grade 3 to 4 events to nearly 60% while dual CTLA-4/PD-1 blockade escalates grade 3–4 events to nearly 60% (40). In CHB, the nonredundant BTLA/SHP 1 circuit operates independently of PD 1/SHP 2 Because the nonredundant BTLA/SHP-1 circuit operates independently of PD-1/SHP-2 (13, 14); therefore, concurrent BTLA and PD 1 targeting may amplify immune activation beyond the threshold tolerated in chronically inflamed livers. This creates two disease specific hazards: immune mediated hepatitis driven by restored cytotoxicity against HBV infected hepatocytes, and HBV reactivation due to transient dysregulation of antiviral immune surveillance, concurrent BTLA and PD-1 targeting may amplify immune activation across both adaptive and innate compartments, potentially exceeding the threshold tolerated in chronically inflamed livers. In addition to the cell-type-specific toxicities outlined above, combination therapy creates two disease-specific hazards in CHB: immune-mediated hepatitis driven by restored cytotoxicity against HBV-infected hepatocytes, and HBV reactivation due to transient dysregulation of antiviral immune surveillance (41). Real world cohorts of HBV-related HCC demonstrate that PD-1 inhibitor therapy can precipitate both hepatitis and reactivation in patients not receiving continuous nucleos(t)ide analogues (NUCs) (42). Conversely, sustained viral suppression with high-barrier NUCs (entecavir or tenofovir) reduces, but does not eliminate, reactivation risk (43).

To mitigate these risks within the proposed Phase I framework, the following safeguards are essential. To mitigate these risks within the proposed Phase I framework, several safeguards are essential. Continuous NUC therapy must be maintained throughout immunotherapy and for at least 6 to 12 months thereafter (44). Exclusion criteria should exclude: active autoimmune disease requiring systemic immunosuppression; Child Pugh B or C cirrhosis or clinically significant portal hypertension; detectable HBV DNA despite NUC therapy; baseline ALT >5×ULN or total bilirubin >2×ULN; prior organ transplantation; and concurrent systemic corticosteroids >10 mg prednisone equivalent daily. Exclusion criteria should exclude: active autoimmune disease requiring systemic immunosuppression, given the theoretical risk that BTLA blockade may exacerbate autoantibody production through dysregulated B-cell and Tfh responses; Child-Pugh B or C cirrhosis or clinically significant portal hypertension, as these patients lack hepatic reserve to tolerate multi-compartment immune-mediated injury; detectable HBV DNA despite NUC therapy; baseline ALT >5×ULN or total bilirubin >2×ULN; prior organ transplantation, because BTLA-HVEM interactions contribute to graft tolerance and their disruption may precipitate rejection; and concurrent systemic corticosteroids >10 mg prednisone equivalent daily. Monitoring comprises ALT, AST, and HBV DNA every 2 to 4 weeks during the first 12 weeks, then every 4 to 6 weeks; additional HBV DNA testing is triggered if ALT exceeds 100 U/L or rises >3× from baseline (45). Dose escalation follows a 3 + 3 design with sentinel dosing in the first cohort. Stopping rules include: (i) dose hold for ALT >5×ULN or any grade ≥3 non-hematologic toxicity; (ii) permanent discontinuation for ALT >3×ULN accompanied by bilirubin >2×ULN (icteric flare), confirmed HBV reactivation (≥1 log_10_ increase in HBV DNA from baseline or HBsAg reappearance), grade ≥4 irAE, or any grade ≥3 irAE persisting >7 days despite corticosteroids; and (iii) trial pause if ≥33% of patients in any cohort experience grade ≥3 hepatic toxicity. A graded management algorithm for hepatic flares is predefined: Grade 1 (ALT >1 to 3×ULN, asymptomatic) continues with weekly monitoring; Grade 2 (ALT >3 to 5×ULN) holds therapy and initiates oral prednisone 1 mg/kg/day; Grade 3 to 4 (ALT >5×ULN, or ALT >3×ULN with bilirubin >2×ULN, or decompensation) permanently discontinues immunotherapy and initiates intravenous methylprednisolone 1 to 2 mg/kg/day, with mycophenolate mofetil or infliximab for steroid-refractory cases (46). Notably, Fc-disabled soluble HVEM fusion proteins may offer a safer modulation strategy by selectively disrupting BTLA/HVEM interactions without engaging FcγR-mediated effector functions, thereby reducing the risk of cytokine release syndrome, thereby avoiding direct killing of HVEM-expressing hepatocytes or stromal cells and reducing the risk of cytokine release syndrome (36–38). Because these decoy receptors preserve HVEM→LIGHT co-stimulatory signaling on antigen-presenting cells, they may maintain partial immunological homeostasis while gradedly attenuating BTLA-mediated suppression, offering a more controllable safety profile for patients with baseline inflammatory markers above the trial median.

Extension to intermediate antigen burden (100 IU/mL < HBsAg < 1000 IU/mL). We recognize that the HBsAg <100 IU/mL subgroup represents a minority of the total CHB population. BTLA-mediated terminal exhaustion operates across the full antigen-load spectrum, not solely in low-antigen patients. In the intermediate stratum (100–1000 IU/mL), higher baseline antigen drives deeper epigenetic exhaustion and more extensive BTLA–SHP-1 circuit fixation, requiring adapted strategies. We propose prolonged NA pretreatment (≥3–5 years) to maximally reduce antigenic stimulation before checkpoint intervention, followed by sequential anti-BTLA monotherapy to dismantle SHP-1–mediated suppression, with cautious addition of low-dose PD-1 blockade only upon demonstration of pharmacodynamic response (e.g., ≥30% reduction in peripheral CD4+ BTLA mean fluorescence intensity or serum sBTLA decline). Because endogenous antigen presentation may be insufficient to sustain restored T-cell responses at this load, co-administration with therapeutic vaccines (e.g., HBV core antigen–targeted T-cell vaccines) should be explored to provide exogenous antigenic fuel. This sequential approach mitigates hepatotoxicity risk while preserving functional cure potential beyond the low-antigen niche.

To enable reproducible translation, we propose an adaptive Phase I concept for NA-suppressed CHB patients within this Tier 1 stratum. Eligibility criteria include HBsAg <100 IU/mL, undetectable HBV DNA on NAs ≥2 years, and elevated peripheral CD4+ BTLA expression. Pending prospective calibration, operational thresholds are proposed as follows: peripheral CD4+ BTLA mean fluorescence intensity at least 2-fold above healthy donor median, or at least 30% of circulating CD4+ or CD8+ T cells BTLA-positive, or serum soluble BTLA at least 2.5 ng/mL, or at least 15% of circulating CD8+ T cells displaying the PD-1+TIM-3+BTLA+ triple-positive phenotype. Patients meeting any two of these four criteria qualify as BTLA-high. Patients are stratified by baseline BTLA tertiles and triple-positive exhausted T-cell frequency. The intervention follows a modified 3 + 3 design starting at 0.3 mg/kg anti-BTLA mAb, selected as one-tenth of the no-observed-adverse-effect level in humanized mouse models, with dose levels at 0.3, 1.0, 3.0, and 10 mg/kg administered intravenously every 3 weeks. Intra-patient escalation is prohibited and cohort expansion to six patients occurs if dose-limiting toxicities occur in no more than one of six patients. Expansion to combination cohorts is governed by pre-specified week-6 decision rules. If at least two patients in a monotherapy cohort experience Grade 3 or higher ALT flare or cytokine release syndrome, expansion is suspended pending safety review. If monotherapy achieves at least 80% BTLA receptor occupancy but fails to reduce triple-positive exhausted T cells by at least 30% or HBsAg by at least 0.5 log_10_ IU/mL, the cohort seamlessly expands to anti-BTLA plus low-dose PD-1 combination with six additional patients. If a subset defined by high baseline soluble BTLA shows disproportionate response, a biomarker-enriched expansion cohort opens for that stratum. Primary endpoints include safety (incidence of grade ≥3 irAEs, ALT flare >5×ULN, HBV reactivation defined as ≥1 log_10_ increase in HBV DNA from baseline or HBsAg reappearance, and hepatic decompensation) and pharmacodynamic markers stratified by mechanistic goal: for terminal exhaustion reversal, at least 50% reduction in PD-1+TIM-3+BTLA+ triple-positive CD8+ T cells and at least 2-fold increase in IFN-γ+TNF-α+ perforin+ CD8+ T cells upon HBV peptide stimulation; for TPEX reinvigoration, at least 50% reduction in TCF1+BTLA+ CD8+ T cells and at least 2-fold increase in TCF1+Ki-67+ CD8+ T cells; for effector differentiation, at least 2-fold increase in IFN-γ+TNF-α+ perforin+ CD8+ T cells upon HBV peptide stimulation; for progenitor expansion, at least 2-fold increase in TCF1+PD-1+CD8+ T cells; for CD4+ helper restoration, at least 2-fold increase in IL-2 production and CD40 ligand (CD40L) expression upon HBV core peptide stimulation; and for microenvironment remodeling, at least 30% reduction in spatial co-localization scores of BTLA with HVEM, PD-L1, and TIM-3 in portal-sinusoidal niches. for CD4+ helper restoration, at least 2-fold increase in IL-2 production and CD40 ligand (CD40L) expression upon HBV core peptide stimulation. In an optional biopsy subset, exploratory microenvironment remodeling endpoints include at least 30% reduction in spatial co-localization scores of BTLA with HVEM, PD-L1, and TIM-3 in portal-sinusoidal niches. Predefined stopping rules for toxicity and biomarker-driven expansion criteria ensure controlled, evidence-based progression. It must be emphasized that no interventional clinical trial of BTLA blockade in NA-suppressed CHB has yet been reported, and the proposed dosing, biomarker thresholds, and combination strategy remain hypothetical. If translatable to human CHB, this approach could expand functional cure rates beyond the ~30% ceiling reported with PD-L1 monotherapy in low-antigen patients (47).

Tier 2: Intermediate antigen burden (100–1000 IU/mL). We recognize that the HBsAg <100 IU/mL subgroup represents a minority of the total CHB population, and BTLA-mediated terminal exhaustion operates across the full antigen-load spectrum. In the intermediate stratum, higher baseline antigen drives deeper epigenetic exhaustion and more extensive BTLA–SHP-1 circuit fixation, requiring adapted strategies. We propose prolonged NA pretreatment (≥3–5 years) to maximally reduce antigenic stimulation before checkpoint intervention, followed by sequential anti-BTLA monotherapy to dismantle SHP-1–mediated suppression, with cautious addition of low-dose PD-1 blockade only upon demonstration of pharmacodynamic response (e.g., ≥30% reduction in peripheral CD4+ BTLA mean fluorescence intensity or serum sBTLA decline). Because endogenous antigen presentation may be insufficient to sustain restored T-cell responses at this load, co-administration with therapeutic vaccines (e.g., HBV core antigen–targeted T-cell vaccines) should be explored to provide exogenous antigenic fuel. This sequential approach mitigates hepatotoxicity risk while preserving functional cure potential beyond the low-antigen niche.

Tier 3: High antigen burden (>1000 IU/mL). At HBsAg levels exceeding 1000 IU/mL, checkpoint blockade alone is unlikely to suffice without prior antigen burden reduction via prolonged NA therapy, siRNA, or HBsAg-targeting agents. This sequencing principle ensures that immune restoration is not overwhelmed by persistent high-level antigen stimulation, which would otherwise deepen exhaustion and increase the risk of hepatotoxicity without proportional functional cure benefit.

## 5 Discussion and outlook

Evidence from 2019 to 2025 positions the BTLA/HVEM axis as a significant contributor to immunosuppression across the spectrum of HBV disease severity, from stable CHB to HBV-ACLF. However, the axis operates in distinct immunological contexts. In stable CHB, it sustains reversible T-cell exhaustion under chronic low-grade antigen stimulation; in ACLF, it drives immune paralysis amid hyperinflammation. Its multiligand topology and cis/trans interactions confer structural leverage; spatial co-localization with other checkpoints amplifies hepatic inhibition; and inflammatory signaling pathways link BTLA engagement to immune paralysis and adverse outcomes. The broad cellular distribution of BTLA across T cells, B cells, NK cells, γδ T cells, dendritic cells, and myeloid populations further reinforces this central position, enabling the axis to orchestrate both innate and adaptive immune tolerance within the liver. Accordingly, biomarker thresholds and interventional strategies validated in ACLF should not be directly extrapolated to the functional cure setting.

Stratified antigen-load framework. To address the full spectrum of CHB, we propose a three-tier antigen-stratified approach. Tier 1 (HBsAg <100 IU/mL): Concurrent low-dose BTLA/PD-1 dual blockade leverages shallow exhaustion and offers the widest safety margin for proof-of-concept trials. Tier 2 (100–1000 IU/mL): Sequential anti-BTLA therapy followed by optional PD-1 addition, potentially combined with therapeutic vaccination, addresses deeper exhaustion while managing hepatotoxicity risk through graded immune restoration. Tier 3 (>1000 IU/mL): Checkpoint blockade alone is unlikely to suffice without prior antigen burden reduction via prolonged NA therapy, siRNA, or HBsAg-targeting agents. This stratified paradigm ensures that BTLA/HVEM-targeted strategies remain clinically relevant across the broader HBV-endemic population, not merely in the most favorable subset. Evidence from 2019 to 2025 positions the BTLA/HVEM axis as a significant contributor to immunosuppression across the spectrum of HBV disease severity, from stable CHB to HBV-ACLF. However, the axis operates in distinct immunological contexts. In stable CHB, it sustains reversible T-cell exhaustion under chronic low-grade antigen stimulation; in ACLF, it drives immune paralysis amid hyperinflammation. Its multiligand topology and cis/trans interactions confer structural leverage; spatial co-localization with other checkpoints amplifies hepatic inhibition; and inflammatory signaling pathways link BTLA engagement to immune paralysis and adverse outcomes. The broad cellular distribution of BTLA across T cells, B cells, NK cells, γδ T cells, dendritic cells, and myeloid populations further reinforces this central position, enabling the axis to orchestrate both innate and adaptive immune tolerance within the liver. Accordingly, biomarker thresholds and interventional strategies validated in ACLF should not be directly extrapolated to the functional cure setting.

From a translational standpoint, BTLA may emerge as a candidate hub for layered, combinatorial, and temporally coordinated strategies rather than a standalone target. Under low antigen load, biomarker-guided limited combination therapies may safely augment HBV-specific immunity toward functional cure. Because BTLA blockade can simultaneously restore T-cell cytotoxicity, Tfh-dependent B-cell cooperation, antibody responses, NK and γδ T-cell innate immunity, and myeloid-cell antigen presentation, it offers a broader portfolio of immune reconstitution than PD-1 monotherapy, which is largely confined to T-cell reinvigoration. Key priorities for the field include harmonizing BTLA quantification assays across prospective cohorts, establishing transferable BTLA-high thresholds calibrated against ALT, AST, HBsAg, and hard clinical outcomes, and integrating cellular, fluid, and tissue biomarkers into deployable companion diagnostic tools with cross-platform consistency.

This Perspective offers a proposed actionable framework tailored to HBV-endemic regions in the Asia-Pacific, where biomarker-guided BTLA-targeted therapies may eventually be integrated into existing NA-based management pathways pending prospective validation. From a translational standpoint, BTLA may emerge as a candidate hub for layered, combinatorial, and temporally coordinated strategies rather than a standalone target. Under low antigen load, biomarker-guided limited combination therapies may safely augment HBV-specific immunity toward functional cure. Because BTLA blockade can simultaneously restore T-cell cytotoxicity, Tfh-dependent B-cell cooperation, antibody responses, NK and γδ T-cell innate immunity, and myeloid-cell antigen presentation, it offers a broader portfolio of immune reconstitution than PD-1 monotherapy, which is largely confined to T-cell reinvigoration. Key priorities for the field include harmonizing BTLA quantification assays across prospective cohorts, establishing transferable BTLA-high thresholds calibrated against ALT, AST, HBsAg, and hard clinical outcomes, and integrating cellular, fluid, and tissue biomarkers into deployable companion diagnostic tools with cross-platform consistency. Accordingly, this Perspective offers a proposed actionable framework for global HBV-endemic regions. Although our cited cohorts derive predominantly from the Asia-Pacific, the immunobiology of BTLA/HVEM-mediated exhaustion is conserved across human populations and viral genotypes, and the minimally invasive sBTLA strategy is applicable to any resource-limited setting, including sub-Saharan Africa, pending local validation. Biomarker-guided BTLA-targeted therapies may eventually be integrated into existing NA-based management pathways worldwide pending prospective validation. We emphasize that safety must remain the overriding priority: the proposed risk mitigation strategies, including continued NUC prophylaxis, strict exclusion criteria, dose escalation safeguards, and predefined stopping rules, are designed to prevent the hepatic flares and reactivation events that could undermine therapeutic gains. It must be emphasized that no interventional clinical trial of BTLA blockade in NA-suppressed CHB has yet been reported, and the proposed dosing, biomarker thresholds, and combination strategy remain hypothetical. Realizing widespread functional cure will require prospective validation in NA-suppressed CHB patients, a critical next step that the hepatology and immunotherapy communities should now pursue.

We emphasize that safety must remain the overriding priority: the proposed risk mitigation strategies, including continued NUC prophylaxis, strict exclusion criteria, dose escalation safeguards, and predefined stopping rules, are designed to prevent the hepatic flares and reactivation events that could undermine therapeutic gains. It must be emphasized that no interventional clinical trial of BTLA blockade in NA-suppressed CHB has yet been reported, and the proposed dosing, biomarker thresholds, and combination strategy remain hypothetical. Realizing widespread functional cure will require prospective validation in NA-suppressed CHB patients, a critical next step that the hepatology and immunotherapy communities should now pursue.

## Data Availability

This Perspective synthesizes previously published studies. No new individual-level data were generated. All cited studies are referenced with DOIs and available through their respective publications. Further inquiries can be directed to the corresponding author.
