# Supplementary material for: The BTLA/HVEM axis as a key regulator for stratified functional cure in chronic hepatitis B
Source: Front Immunol. 2026 Aug 10;17:1882879. doi: 10.3389/fimmu.2026.1882879 (PMC13500586; doi:10.3389/fimmu.2026.1882879)
Supplement: Supplementary file 1 [file Table1.docx]

Table S1. Stratified pharmacodynamic endpoints aligned with mechanistic goals in the proposed BTLA-targeted Phase I trial for NA-suppressed chronic hepatitis B

| Mechanistic goal | Primary immune compartment | Proposed intervention | Key pharmacodynamic endpoint | Quantitative target |
| --- | --- | --- | --- | --- |
| Reversal of terminal exhaustion | CD8+ effector T cells | Anti-BTLA mAb | Reduction in PD-1+TIM-3+BTLA+ triple-positive CD8+ T cells; increase in IFN-γ+TNF-α+ perforin+ CD8+ T cells | ≥50% decrease; ≥2-fold increase |
| Expansion of progenitor exhausted T cells | Stem-like CD8+ T cells | Anti-BTLA mAb ± low-dose PD-1 | Increase in TCF1+PD-1+CD8+ T cells | ≥2-fold increase |
| Restoration of CD4+ helper function | CD4+ T follicular helper cells | Anti-BTLA mAb | Increase in IL-2 production and CD40L expression upon HBV core peptide stimulation | ≥2-fold increase |
| Reshaping of intrahepatic microenvironment | Portal-sinusoidal niche | HVEM-Fc (Fc-disabled) | Reduction in spatial co-localization scores of BTLA/HVEM with PD-L1/TIM-3; decrease in hepatic ISG15/CXCL10 signature | ≥30% reduction |
